# Inter-Species Rescue of Mutant Phenotype—The Standard for Genetic Analysis of Human Genetic Disorders in *Drosophila melanogaster* Model

**DOI:** 10.3390/ijms23052613

**Published:** 2022-02-27

**Authors:** Alexandru Al. Ecovoiu, Attila Cristian Ratiu, Miruna Mihaela Micheu, Mariana Carmen Chifiriuc

**Affiliations:** 1 Department of Genetics, Faculty of Biology, University of Bucharest, 060101 Bucharest, Romania; alexandru.ecovoiu@bio.unibuc.ro; 2 Department of Cardiology, Clinical Emergency Hospital of Bucharest, 014461 Bucharest, Romania; miruna.micheu@yahoo.com; 3 The Research Institute of the University of Bucharest and Faculty of Biology, University of Bucharest, 050095 Bucharest, Romania; carmen.chifiriuc@bio.unibuc.ro

**Keywords:** human genetic disorder, *Drosophila melanogaster* model, heterologous rescue, functional complementation, genetic analysis

## Abstract

*Drosophila melanogaster* (the fruit fly) is arguably a superstar of genetics, an astonishing versatile experimental model which fueled no less than six Nobel prizes in medicine. Nowadays, an evolving research endeavor is to simulate and investigate human genetic diseases in the powerful *D. melanogaster* platform. Such a translational experimental strategy is expected to allow scientists not only to understand the molecular mechanisms of the respective disorders but also to alleviate or even cure them. In this regard, functional gene orthology should be initially confirmed *in vivo* by transferring human or vertebrate orthologous transgenes in specific mutant backgrounds of *D. melanogaster*. If such a transgene rescues, at least partially, the mutant phenotype, then it qualifies as a strong candidate for modeling the respective genetic disorder in the fruit fly. Herein, we review various examples of inter-species rescue of relevant mutant phenotypes of the fruit fly and discuss how these results recommend several human genes as candidates to study and validate genetic variants associated with human diseases. We also consider that a wider implementation of this evolutionist exploratory approach as a standard for the medicine of genetic disorders would allow this particular field of human health to advance at a faster pace.

## 1. Introduction

Advances in animal model-based research markedly increased our understanding of molecular mechanisms that regulate physiological and pathological processes. Perhaps one of the greatest achievements in this field is the development of genetically engineered animal models which offer a valuable platform for disease modelling and testing of potential therapeutic strategies. Accordingly, choosing reliable animal models represents a critical step to speed up the successful integration of precision medicine into daily clinical practice [1]. With its short generation time, low cost, large brood size and ease of genetic manipulation, *Drosophila melanogaster* (the fruit fly) has emerged as a key organism to explore disease-related genetic mechanisms [2].

*Homo sapiens* and *D. melanogaster* share strong similarities regarding many biological functions such as reproduction, embryo development, locomotion, respiration, circulatory system and neurodevelopment [3,4,5]. This relies on a high degree of evolutionary conservation of important genomic features such as genes, core regulatory mechanisms and genetic pathways. 

These analogies endorse phenotypic rescue experiments conceived to reveal inter-species functional gene orthology. A crucial rescue assay for modelling a human genetic disorder (hGD) in *D. melanogaster* is the functional complementation (heterologous rescue) of an appropriate mutant fruit fly strain by the orthologous human or mammalian transgene associated with the respective hGD. If the two genes or proteins with similar nucleotide or amino acid sequences are also functionally related, namely, if the molecular functions are evolutionary conserved, the human wild-type allele (hWT) of the gene of interest (hGOI) is expected to rescue, at least partially, the fruit fly mutant phenotype. In other words, to rescue or save a phenotype means to restore it to wild type with a transgenic copy of the orthologous gene from other species.

In the technical jargon, various synonyms are used for the inter-species rescue of the mutant phenotype. For example, scientists working with a humanized yeast model (*Saccharomyces cerevisiae*) coined the terms cross-species functional complementation, standing for heterologous rescue, and heterologous expression, meaning the ectopic expression of a human gene in yeast strains, regardless of whether the yeast is wild type or mutant [6,7]. Herein, we conversely utilize the terms heterologous rescue and functional complementation used by FlyBase [8], but the more intuitive phrase phenotype rescue (such as lethality rescue) is also used whenever appropriate. The rescue term is also used for saving a fruit fly mutant phenotype with a dWT (*Drosophila* wild-type) transgene to confirm that the phenotype is indeed determined by the presumed gene and not by a hidden mutation present in the genetic background. We will further refer the phenotype rescue with dWT as intra-specific rescue, to differentiate from inter-specific, heterologous rescue or functional complementation equivalent terms. A different type of rescue is the chemical rescue, which is not a genetic one but instead is part of an endeavour to find chemicals able to alleviate or save mutant phenotypes, allowing the screening for potential new drugs.

*D. melanogaster* is suitable for different modelling approaches of human genetic diseases. One strategy involves targeted mutagenesis of *Drosophila* gene of interest (dGOI) in conserved sequences shared with hGOI, or RNAi inactivation of dGOI, to reproduce phenotypes resembling pathologic aspects of the hGD in *D. melanogaster*. An alternative is the replacement of dGOI with a disease-specific allele of hGOI to mirror clinical phenotypes in *D. melanogaster*. Last but not least, a different avenue is to introduce into fruit flies either a wild-type or a mutant copy of a hGOI having no evident structural ortholog in the *D. melanogaster* genome, but which may be useful to reproduce *in vivo* some molecular interactions important for understanding of the hGD. Whichever experimental alternatives are to be considered in practice, either individually or overlapping, a key step is to perform preliminary inter-specific phenotype rescue experiments, namely, to check if the wild-type copy of hGOI is able to functionally compensate a mutant allele of the orthologous dGOI. A positive result shows evolutionary functional conservation between the two species and reinforces *D. melanogaster* as a suitable experimental platform for modelling that particular hGD. On the other hand, a failure of the inter-specific phenotype rescue attempt, either an intrinsic or a false negative one, may induce the geneticists to decide on a mammalian model.

Since the experimental strategies used to model hGDs on *D. melanogaster* are already detailed in a few excellent papers [9,10], we choose to focus on various examples of inter-species phenotype rescues relevant for the medical research.

High-quality sequenced and assembled genomes have become increasingly available and allow experts to identify genes and regulatory sequences relevant for human medical research. This achievement relies on a deep comparative scanning of the two genomes with state-of-the-art bioinformatics tools. If structural orthologous gene pairs of interest are identified, targeted mutagenesis may be induced in *D. melanogaster* by an array of highly effective methods developed for this experimental model. The mutant alleles are then subjected to genetic analysis methods to check for functional orthology to human genes responsible for the aberrant phenotypes underlying the respective hGD. 

**Figure 1:**
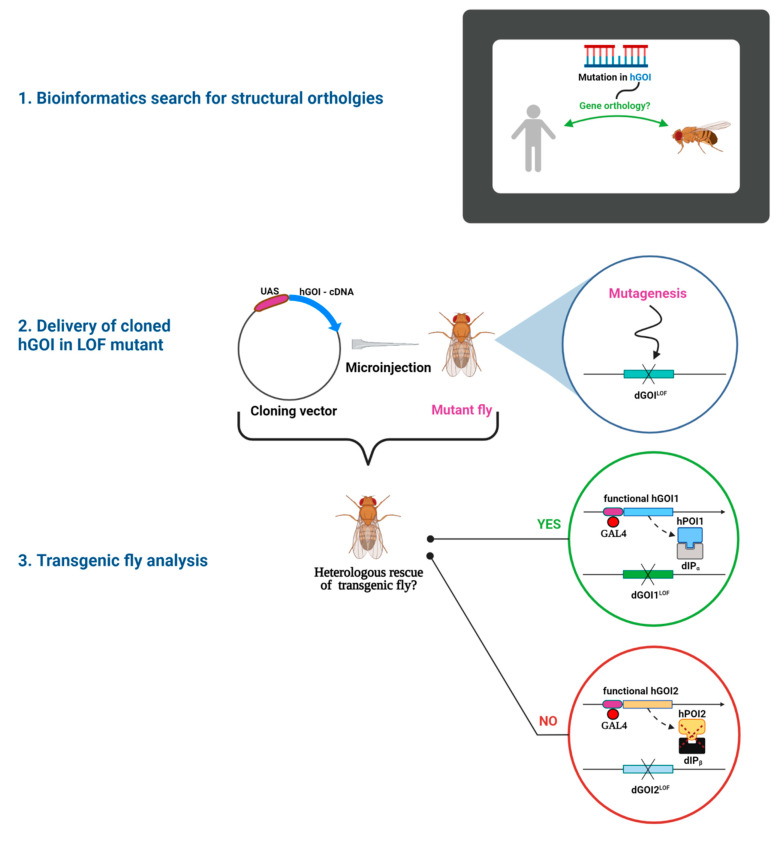
General outline of the heterologous rescue of *D. melanogaster* mutant phenotypes determined by lethal LOF alleles of dGOIs with transgenic cDNAs corresponding to the orthologous hGOIs associated with a hGD. (1) Bioinformatics analysis is deployed to search in the fruit fly genome for an orthologous gene for a human gene associated with a genetic disorder. (2) A cDNA copy of the wild-type allele of hGOI is cloned into an insertional vector under the control of a UAS enhancer sensitive to GAL4 activator. The UAS–cDNA construct is delivered by micro-injection into mutant embryos heterozygous for dGOI^LOF^ allele (obtained by targeted mutagenesis), and the resulting transgenic adults are subsequently crossed with a heterozygous dGOI^LOF^ fruit fly strain able to produce the GAL4 activator. (3) Transgenic analysis of descendant dGOI^LOF^/dGOI^LOF^ flies that contain a functional hGOI activated by GAL4 may reveal two distinct situations. YES (complete or partial heterologous rescue). The functional hGOI1 encodes a human protein of interest (hPOI1) which is able to properly interplay with an interacting protein (dIPα) in *D. melanogaster* molecular background; since dIPα is the proximal interactor of the normal protein encoded by wild-type copy of dGOI1, the correct interaction between hPOI1 and dIPα rescues lethality of LOF/LOF transgenics. NO (heterologous rescue fails). On the other hand, the human protein hPOI2, encoded by a different hGOI2 transgene, does not interact accurately with dIPβ and the heterologous rescue fails. Created with BioRender.com (accessed on 23 February 2022).

Briefly, the modus operandi of this experimental approach is the identification of a human gene associated with the hGD of interest, bioinformatics comparative analysis to scan *D. melanogaster* genome for a candidate structural ortholog of the human gene, generation and analysis of relevant mutant alleles in *D. melanogaster*, delivery of the orthologous human cDNA into the appropriate fruit fly mutant background by means of effective molecular constructs and checking for partial or complete rescue phenotype of the transgenic fruit flies. Commonly, the heterologous rescue experiments target mutant phenotypes determined by loss-of-function (LOF) alleles, which are either hypomorphic alleles with reduced activity or null alleles with no residual activity [11]. The experimental steps of heterologous rescue, which rely on the modular and versatile UAS-GAL4 system, are outlined in Figure 1 [12,13]. 

As an example, if the LOF allele is a recessive null lethal one, very young heterozygous mutant embryos are microinjected with an insertional vector containing the orthologous hGOI cloned under an UAS enhancer control. Transgenic adults containing both the LOF allele and UAS–hGOI construct are crossed with a strain containing both the LOF allele and a GAL4-driver with either specific or generic pattern of expression. If, in the F1 generation of this cross, the LOF/LOF homozygous individuals are viable, the heterologous rescue was successful due to activation of UAS-hGOI by GAL4. Therefore, the structural ortholog’s genes are also functionally orthologous, indicating that at least some of their functions were conserved during evolution.

A successful heterologous rescue result is a very strong indicator for functional orthology between the members of human–fruit fly gene pairs. It is important to mention that any functional improvement of the mutant phenotypes of LOF flies such as rescue of lethality, proceeding through a later developmental stage, increased lifespan, increased fertility, improved behavior, etc., deserves attention and qualifies the functionally rescued dGOI as an attractive candidate for modeling hGDs in *D. melanogaster* [14]. Even when heterologous rescue of mutant fruit flies was not performed with a hWT but with a mammalian orthologous gene [15,16], this functional conservation is a strong genetic logic to start a research project on that gene model [14,17].

FlyBase reports the rescue experiments as “heterologous rescue” in the Overview tab of the report of a human genetic disorder modelled in *D. melanogaster*. The link to the respective fruit fly orthologous gene opens a Gene Report webpage which contains a Functional Complementation Data Table. If functional data are available, links under the Ortholog tabs showing functional complementation and Supporting References are present. In practice, many studies reported in FlyBase describe rescuing of abnormal phenotypes induced by RNAi suppression of GOI, but care should be taken when interpreting such data. A recent report dealing with the difficulties arising from the RNAi method reveals that residual functional activity of some genes in *D. melanogaster* still exists even when this technology is improved [18]. 

We reviewed data obtained from heterologous rescue experiments supporting human–fruit fly functional gene equivalence and their value for genetic analysis of hGDs. To this end, we present relevant examples of neurodegenerative and neuromuscular disorders, cardiac pathophysiology, cancer and infectious diseases. To our best knowledge, the present paper is the first attempt to scrutinize up-to-date scientific literature and FlyBase (FB2021_06) for the vast majority of the heterologous rescue experiments performed in *D. melanogaster*. We argue that preliminary experiments of mutant phenotype rescue should be the paradigm for any relevant genetic analysis of hGDs on the *D. melanogaster* model.

## 2. Neurodegenerative and Neuromuscular Disorders

For more than 20 years, *D. melanogaster* has been employed to tackle neurodegenerative and neuromuscular human afflictions [19,20]. Due to its relatively complex brain, which harbors around 300,000 neurons organized into specialized areas with discrete functions [21], *D. melanogaster* displays complex behaviors such as learning, memory, depression, anxiety, competitiveness, aggressiveness and alcoholism. Consequently, *D. melanogaster* represents a valuable system for the study of neuronal dysfunction and related disorders particular to several neurodegenerative diseases such as Alzheimer’s disease (AD), amyotrophic lateral sclerosis (ALS), Angelman’s syndrome (AS), autism spectrum disorder (ASD), Charcot–Marie–Tooth (CMT) disease, Friedrich’s ataxia (FA) and Parkinson’s disease (PD), to name a few [22]. 

### 2.1. Parkinson’s Disease

PD is one of the most common neurodegenerative diseases, accompanied by specific tremors and slow movement caused by degradation of dopaminergic (DA) neurons in the midbrain. To decipher the poorly understood mechanisms of selective degeneration of DA neurons, interactions between the products of human *α-Synuclein* (*α-Syn*), *parkin RBR E3 ubiquitin protein ligase* (*PRKN*) and *PAELR* genes were modelled in *D. melanogaster* brain neurons [23]. Co-expressing *PRKN* and *α-Syn* in transgenic flies rescued the loss of DA neurons and reduced the aggregation of α-*Syn*, a mutant phenotype, which endogenous *parkin* (*park*) from *D. melanogaster* was not able to rescue in *α-Syn* transgenic flies. This example can be viewed as a type of phenotypic rescue reflecting a putative incapacity of *Drosophila*’s *park* gene product to interact with human α*-Syn*.

On the same topic, Burchell et al. [24] showed that overexpression of human *Fbxo7* gene, associated with a severe form of autosomal recessive early-onset PD [25], significantly rescued several mutant phenotypes such as locomotor defects, DA neuron loss and muscle degeneration determined by LOF of *parkin*. Pathogenic mutant *Fbxo7* alleles were not able to rescue the loss of *park*, a result reinforcing the notion that the corresponding proteins share a common role in mitochondrial maintenance and mitophagy. 

Mutations in *coiled-coil-helix-coiled-coil-helix domain containing 2* (*CHCHD2*) human gene negatively impact the oxidative phosphorylation processes in mammalian cells [26] and are associated with an autosomal dominant form of late-onset PD. In *D. melanogaster*, LOF and hypomorphic *Chchd2* alleles affect the maintenance of mitochondrial crista structure and lead to neuronal phenotypes associated with PD, such as sensitivity to oxidative stress, motor dysfunction, short lifespan and loss of DA neurons with age [27]. Expression of either transgenic dWT *Chchd2* or hWT *CHCHD2*, but not missense alleles of the latter, successfully rescued the mitochondrial morphology and DA neurons loss induced by hypomorphic *Chchd2^H43^* in *D. melanogaster*.

Another study concerning PD [28] focuses on exploring a specific subset of human *iPLA2-VIA/PLA2G6* mutations that direct α-*Syn* aggregation and DA neurodegeneration specific for the PARK14-linked PD with α-synucleinopathy. The *iPLA2-VIA/PLA2G6* gene codifies for an enzyme that is fundamental to phospholipids synthesis by the remodeling pathway or Lands’ cycle [28]. *IPLA2-VIA*-null allele impacts the early developmental stages of *Drosophila* mutants and leads to alterations of neurotransmission and midbrain DA neurons’ degeneration, causing gradual locomotor defects and sleep disruption [28]. When transgenic hWT *iPLA2-VIA* was expressed in the neurons of mutant flies, the motor and paralytic phenotypes were rescued, pointing to functional conservation between the two orthologous genes.

In addition to the previous examples, complex molecular interactions characterizing PD, such as the imbalance in trace metal levels characteristic for some forms of PD and AD, may be also addressed. The *metal-responsive transcription factor 1* (*MTF-1*) gene is evolutionary conserved between *D. melanogaster* and mammals [29] and counteracts the effects of heavy metal loads. In mammals, *MTF-1* was found to induce transcription of specific target genes in response to oxidative stress and infection [30]. A study focusing on the interactions between metal homeostasis and *park* function established that mutants expressing both *park* and *MTF-1* LOF alleles in homozygous condition define a genetic assembly termed synthetic lethality [31]. The introduction of a transgene of *MTF-1* in the double homozygous *park* and *MTF-1* mutants rescued the lethality and has significantly increased the lifespan of *park* homozygous mutants. Alternatively, human *MTF-1* has been able to rescue the short lifespan phenotype of *park* mutants [31], and largely, but not completely, rescued the metal sensitivity characterizing the LOF *MTF-1* flies [32].

Loss of function *pink1* mutant flies experience PINK1 deficiency and display motor disturbances as well as corrupted function of Complex I of the mitochondrial respiratory chain, and thus increased sensitivity to apoptotic stress. In humans, mutations in *PTEN (phosphatase and tensin homologue)-induced kinase1* (*PINK1*) are strongly correlated with recessive forms of PD. HWT allele, but not mutant *PINK1*, rescued the phenotype exhibited by LOF *Pink1* mutant flies [33].

### 2.2. Amyotrophic Lateral Sclerosis

ALS is arguably the most prevalent motoneuron disorder that leads to fatal adult-onset neurodegenerative progression [34,35]. The genetic basis of ALS overlaps at least partially with that of frontotemporal dementia, and often, the ALS patients concurrently develop cognitive and behavioral alterations [36,37].

Among over 30 genes that harbor ALS causing mutations, some of the most noticeable are superoxide dismutase-1 (SOD1), chromosome 9 open reading frame 72 (C9orf72), fused in sarcoma (FUS) and TAR DNA-binding protein (TARDBP) [38].

Various studies focused on validating the effects of human *SOD1*, as well as of other ALS associated genes, were performed on the fly phenotype in order to establish an ALS experimental model, as reviewed elsewhere [38,39,40]. In *D. melanogaster* it has been shown that null alleles of resident *Sod1* determine impaired locomotor activity and lethality. These severe phenotypes are fully rescued by *SOD1^WT^* but not by its clinically relevant mutant alleles such as *SOD1^A4V^*, *SOD1^G37R^* or *SOD1^I113T^* [41], which confirm the impaired functions of these alleles in human patients. In addition, it was revealed that even a localized adult motor neuron expression of *SOD1^WT^* restored the lifespan of null *Sod1*-null flies to 60% of the normal controls [42].

In humans, both *TARDBP* and *FUS* code for DNA- and RNA-binding proteins involved in RNA processing of thousands of targets and share some common functionality underlined by similar pathogenic outcomes stemming from mutations [43,44,45]. *D. melanogaster* harbors *TAR DNA-binding protein-43 homolog* (*TBPH*) and *cabeza* (*caz)* as orthologs of *TARDBP* and *FUS*, respectively. LOF alleles of *TBPH* cause the disruption of mitochondrial trafficking accompanied with severe motor dysfunctions revealed by low rates of eclosion, altered larval crawling and adult climbing capacity [46]. Except the adult climbing mutant phenotype, all of the others are fully rescued by expressing either *TBPH^WT^* or *TARDBP^WT^* transgenes. Intriguingly, the mitochondrial transport defects were also rescued by expressing *TARDBP^M337V^*, an ALS-linked allele. This particular example of phenotypic rescue reveals that the pathogenic variant can display normal function in *D. melanogaster*, indicating that this allele is not involved in ASL. The same study found that *caz^1^*-null mutants [47] presented a significant decrease in mitochondria and vesicle transport. These phenotypes were rescued by expressing *FUS^WT^* in mutant flies, but the *caz^WT^* transgene was able to fully rescue both phenotypes only at 29 °C, when it is overexpressed, and only partially at 25 °C. Surprisingly, the *FUS^P525L^* pathogenic allele as well as its equivalent *caz^P938L^* successfully rescued the mitochondrial transport defects but not the vesicle transport. Regardless of expressing WT or pathogenic *FUS* alleles, other phenotypes particular to *caz^1^* mutants such as eclosion, larval crawling and climbing defects were fully rescued. To cement the functional overlap between *TBPH* and *caz*, the reduced viability, lifespan, eclosion and climbing ability of *TBPH* mutants were fully rescued by neuronal overexpression of *caz*, but not *vice versa*. Overexpression of *caz^WT^* did not rescue the mitochondrial transport defects or larval crawling impairment. Consistent with these findings, it was previously shown that *TARDBP^WT^* transgene is also able to rescue the drastically reduced locomotor speed of mutant flies lacking TBPH, just as overexpressing *caz^WT^* [47].

### 2.3. Autism Spectrum Disorder

ASD comprises of complex developmental conditions and it is mainly characterized by behavioral symptoms such as impaired communication skills, defective social interaction, repetitive behavior, limited capacity to live independently, etc. but also by a high prevalence of gastrointestinal problems such as diarrhea, constipation, vomiting, and abdominal pain, just to mention a few [48,49,50,51,52].

SFARI Gene (https://gene.sfari.org/, accessed on 20 December 2021) is a database indexing the genes associated with ASD and categorizes these genes according to evidence of their involvement in ASD [53]. Within Category 1 of high confidence for implication in ASD, there are currently 207 genes, which are also present in other similar gene lists or were previously identified in an extensive exome sequencing study [54]. Out of these, 203 have orthologs in *D. melanogaster*, with 141 genes having a *Drosophila* RNAi Screening Center integrative ortholog prediction tool (DIOPT) score of at least of 0.6 [55]. DIOPT scores are provided by the DIOPT integrative tool [56], which is currently at version 8.5 and enables the search of orthologs in different species among the data provided by 18 large-scale ortholog prediction tools. The aforementioned list of orthologs with conclusive DIOPT scores includes the *D. melanogaster* genes *Fmr1*, *Pten* or *ubiquitin protein ligase E3A* (*Ube3a*), with DIOPT scores of 0.73, 0.87 and 0.93, respectively [55].

In *D. melanogaster*, *Fmr1* gene is a structural ortholog of *FMRP translational regulator 1* (*FMR1*) human gene. Mutations in *FMR1* gene are causing human fragile X syndrome (FXS), which is probably the most common heritable foundation of autism disorders and mental retardation [57]. The two paralogs of *FMR1*, *FMR1 Autosomal Homolog 1* (*FXR1*) and *FXR2*, also share a strong sequence similarity with *Fmr1*, thus making it difficult to choose a certain gene for testing the functional orthology. The *Fmr1^50M^*-null allele determines a wide range of mutant phenotypes, the most striking in neurons and germ cells of adult flies. All three paralog human genes were tested in order to assess whether mutant phenotypes can be rescued in *Fmr1*-null mutants. Targeted neuronal expression of both hWT *FMR1* and dWT *Fmr1* transgenes rescued the characteristic FXS phenotypes such as higher brain protein levels, abnormal circadian rhythm patterns determined by small ventrolateral neurons’ synaptic arbor overgrowth defect and increased synaptic branching of neuromuscular junction. Expression of *FXR1* and *FXR2* failed to rescue the neuronal mutant phenotypes, however, all three human paralogs were equally competent to overcome non-neuronal symptoms in *Fmr1* mutants such as severely reduced fertility caused by immotile sperm exhibiting defects in sperm tail microtubule organization [58]. These results highlight that care should be taken when testing structurally similar functional orthology candidates, especially when working with genes that manifest both evolutionary conserved and shared roles.

The *PTEN* gene has tumor suppressor activity, and even a partial loss of PTEN activity leads to cancers [59] or PTEN hamartoma syndrome, consisting of a variety of disorders such as macrocephaly, epilepsy, mental retardation and ASD [60,61]. In mammals as well as in *D. melanogaster*, PTEN, which is a dual lipid and protein phosphatase, is a critical repressor of phosphoinositide 3-kinase (PI3K)/protein kinase B (PKB/AKT) pathway [62]. In *D. melanogaster*, *Pten^100^*/*Pten^117^* represents a strong hypomorphic heteroallelic combination that leads to increased larval growth and, consequently, increased pupal volume and adult weights. Expression of the *PTEN^WT^* allele in embryos and larvae successfully rescued the hypomorphic mutants [63]. Within the same study, the authors devised a scalable experimental platform to functionally test about 100 human *PTEN* alleles with potential clinical relevance. To do this, they overexpressed an activated *Phosphatidylinositol 3-kinase 92E* (*Pi3K92E*) allele (*PI3K92E–CAAX–PI3K^act^*) in the wing imaginal disc, resulting in adults harboring enlarged wings. This phenotype was rescued by *PTEN^WT^* but not by *PTEN^C124S^*, which lacks both protein and lipid phosphatase activity, or by the *PTEN^G129E^* lipid phosphatase dead alleles. *PTEN^Y138L^*, which is deficient for protein phosphatase activity, is able to partially alleviate the wing size phenotype. These either failed or partially successful heterologous rescue experiments demonstrated that PTEN-dependent suppression of PI3K/AKT tissue growth in *Drosophila* is dependent upon both lipid and protein phosphatase activities. Using this wing size-based system, they successfully tested the functionality retained by the PTEN alleles by scoring their ability to partially or completely rescue the oversized fly wing.

Mutations affecting *UBE3A* gene are the main cause for AS, a relatively common human disorder involving aberrant central nervous system development and characterized by mental retardation and locomotor impairment [64]. In our laboratory, we obtained a new *Ube3a* allele, symbolized as *As^m1.5-R^*, consecutive to transposon-mediated mutagenesis [65]. This allele is semilethal for mutant homozygous males, which, when surviving to adulthood, elicit decreased locomotor performances. We successfully rescued this abnormal phenotype in flies raised for more than a year on culture medium supplemented with omega-3 polyunsaturated fatty acids, i.e., eicosapentaenoic and docosahexaenoic acids [66]. A true heterologous rescue was demonstrated for the learning abilities of both larvae and adult *Ube3a*-null mutants [67]. Individuals from both mutant categories exhibited impaired learning abilities as scored by using the aversive phototaxis suppression assay, which tests the ability of fruit flies to link an unpleasant taste stimulus with light. Expressing *UBE3A^WT^* transgene by the pan-neuronal elav-GAL4 driver in the mutant background rescued the mutant learning defects.

Altogether, the previous rescue and disease modelling examples reinforce the power of the *D. melanogaster* experimental model. In addition, many other mammalian genes associated with neurodegeneration-related diseases were studied in the fly model, as presented in Table 1.

## 3. Cardiac Disorders

Several key characteristics related to cellular processes, signalling pathways and gene conservation endorse *D*. *melanogaster* as a model of choice for studying human cardiac development, function and diseases. First, although seemingly simplistic, the fruit fly circulatory system, consisting of a tube-like heart that pumps the haemolymph, shows developmental and functional similarities to the vertebrate heart [134,135,136,137]. Second, both organisms (*D. melanogaster* and *H. sapiens*) share some regulatory cardiogenic networks encompassing critical cardiac transcription factors such as *tin*/*Nkx2.5*, *Mef2*/*Mef2C*, *pannier*/GATA family and *Hand*/*HAND1* and *HAND2*, which are required for cardiac progenitor specification [137,138,139,140,141,142,143]. Third, there is a strong gene conservation with human genes, particularly with disease-related genes [144]. By performing a systematic BLAST analysis of 929 human disease gene entries associated with at least one variant in the Online Mendelian Inheritance in Man (https://www.omim.org/, accessed on 18 December 2021) database against the reference sequence of *D. melanogaster,* Reiter and colleagues revealed that 77% of disease genes queried had *Drosophila* counterparts [144]. Of note, 26 of them were associated with various cardiovascular diseases such as cardiomyopathies, hypertension and conduction defects [3].

### 3.1. Congenital Heart Defects

Congenital heart defects (CHDs) are the most common birth disorders, affecting 0.8% to 1.2% of live infants [145]. Although it is largely acknowledged that genetic factors are strongly involved in CHD pathogenesis, the great majority of responsible genes remain elusive [146]. *Drosophila*-based research enabled the recognition of new CHD-related genes. Zhu et al. [147] developed a *Drosophila*-based functional system to rapidly and efficiently screen large numbers of candidate genes detected in patients with severe CHDs. By using heart-specific RNAi silencing, they tested 134 genes, of which more than 70, including a subgroup encoding histone modifying proteins, were found to be essential for the development, structure and function of the fruit fly heart. The silencing of genes responsible for H3K4 and H3K27 methylation (i.e., *kis*/*CHD7*, *wds*/*WDR5*, *Trx*/*MLL2*) caused developmental lethality (up to 84%) and severe structural heart anomalies and reduced adult longevity. Moreover, a gene substitution strategy comprising concurrent heart-specific silencing of the fly gene homolog and expression of either a wild-type variant or a pathogenic one was applied to validate the role of these genes in CHDs. As a proof-of-concept, the authors explored the potential of the *WDR5^WT^* human allele to rescue the pathogenic phenotype generated by silencing of the endogenous *wds Drosophila* homolog. *WDR5^WT^* overexpression significantly reduced developmental lethality and restored abnormal heart morphology, as opposed to the CHD patient-derived *WDR5^K7Q^* mutant allele, which resulted in similar pathogenic cardiac manifestations. 

### 3.2. Cardiomyopathy Phenotypes

Cardiomyopathies are a heterogeneous group of myocardial diseases in which the cardiac muscle is structurally and functionally abnormal, often due to a genetic cause [148]. The injury can be limited to the heart or part of a generalized systemic disorder; either way, the genetic architecture is very diverse [149,150]. 

Hypertrophic cardiomyopathy (HCM) is the most prevalent inherited cardiomyopathy, affecting at least 1 in 500 individuals in the general population [151,152]. The underlying genetic etiology is complex, mainly involving variation in sarcomeric or sarcomeric-related genes, but mutation in other genes can cause similar phenotypes comprising left ventricular hypertrophy (LVH). Of the 57 candidate genes included in diagnostic HCM gene panels, only 8 have been nominated as having definitive evidence, including myosin light chain 2 (*MYL2*) [153,154].

Recently, Manivannan and colleagues identified a novel recessive frameshift variant in *MYL2* (p.Pro144Argfs*57) resulting in early-onset HCM and death in infancy [155]. A fly model was used to demonstrate that p.Pro144Argfs*57 variant was in fact a LOF allele. The expression of the *Drosophila* ortholog *Mlc2* was knocked down using transgenic RNAi lines, which led to multiphasic lethality, with progenies dying before the pupal stage, and impaired systolic function. Both the developmental lethality and cardiac dysfunction were partially rescued by *MYL2^WT^* but not by the frameshift variant. The incomplete restoration of fly phenotype was most likely due to sequence differences between the two organisms, given that the *Mlc2* N-terminal region has additional sequences that are required for its function [156].

An HCM-like phenotype can be encountered in other conditions involving LVH, such as FA, which is a neurodegenerative disease caused by a GAA trinucleotide repeat expansion in *frataxin* gene (*FXN*) [157,158]. Over time, the cardiomyopathy potentially progresses to a dilated form. It has been shown in *D. melanogaster* that RNAi-mediated *frataxin* (*fh*) depletion prompted enlargement of cardiac diameters and reduction in systolic function, which were fully rescued by complementation with *FXN^WT^* human allele [159].

Gonçalves et al. [160] reported a study concerning three human families A, B and C affected by mutations in *adducin 3* (*ADD3*) gene encoding for adducin-γ, associated with various disabilities such as intellectual disability, microcephaly, cataracts and skeletal defects. Patients from family A are also homozygous for a missense mutation of *lysine acetyltransferase 2B* (*KAT2B*) and the expanded pathologic spectrum, including cardiomyopathy and renal problems. This allele is symbolized as *KAT2B^F307S^* and determines the substitution of highly conserved Phe with Ser at position 307 of the human protein. These genetic disorders are prone to be modeled on *D. melanogaster*, since the fruit fly genome contains the *hu li tai shao (hts)* ortholog for *ADD1* (*adducin-α*), *ADD2* (*adducin-β*) and *ADD3*, and *Gcn5 acetyltransferase* (*Gcn5*) paralog for *KAT2A* and *KAT2B*.

The *hts^null^* hemizygous flies die as late larvae and only a few impaired, short living escapers reach the adult stage. Expression of *ADD3^WT^* does not rescue viability, but the ubiquitous co-expression of *ADD1^WT^* and *ADD3^WT^* leads to heterologous rescue by increasing the number of viable adults. Alternatively, *ADD1^WT^*/*ADD3^E659Q^* co-expression (where *ADD3^E659Q^* is a human mutant allele reported for family A) induces only a partial rescue of *hts^null^* hemizygous flies, revealing that *ADD3^E659Q^* behaves as a hypomorphic allele in the fruit fly mutant background. The *hts* mutant flies rescued by *ADD1^WT^*/*ADD3^E659Q^* did not show any significant differences in heart period, cardiac output, fractional shortening and arrhythmia index as compared to those rescued with *ADD1^WT^*/*ADD3^WT^*.

The genetic analysis of an allele of *Gcn5* gene in *D. melanogaster* equivalent to *KAT2B^F307S^* supports the hypothesis that *KAT2B* is associated with heart and kidney mutant phenotypes in humans. Specifically, the *Gcn5^E333st^* allele, also referred as *Gcn5^null^,* is lethal in hemizygous individuals, which arrest development at the late larval to early pupal stage [161]. When *KAT2A^WT^* and *KAT2B^WT^* are expressed either individually or synchronously in hemizygous Gcn5*^null^* flies, the rescue of transgenic flies fails, suggesting that these orthologous genes have functionally diverged during evolution. As expected, transgenic hemizygous *Gcn5^null^* flies appear to be completely rescued by the *Gcn5^WT^* allele, but partially rescued by the *Gcn5^F304S^* allele, as lethality still often occurs in pupae or in early adults and the escapers exhibit morphological impairments. *Gcn5^F304S^* resembles *KAT2B^F307S^* and encodes a protein variant having Ser instead of Phe at position 304. Remarkably, the escapers expressing *Gcn5^F304S^* not only have obvious morphological mutant phenotypes, but also present a prolonged heart period and reduced cardiac output comparative to both a control strain and *Gcn5^null^* hemizygous flies rescued with *Gcn5^WT^*. 

RNAi silencing of *Gcn5* (*Gcn5^RNAi^*) in *D. melanogaster* induces functional heart problems, while silencing of *hts* (*hts^RNAi^*) does not. However, silencing of both genes in *Gcn5^RNAi^* and *hts^RNAi^* flies aggravates heart period length and the arrhythmia index induced by *Gcn5* knockdown alone. These data reveal that *Gcn5* is directly involved in heart function in *D. melanogaster*, while some *hts* mutations increase the severity of the *Gcn5*-null phenotype [160], spotting *hts* as a potential genetic enhancer of *Gnc5*.

Such complex heterologous rescue experiments of specific *D. melanogaster* mutants simulate patients with multilocus genetic diseases, affected by pathogenic mutations located in more than one gene. In this case, simultaneous knockdown of *hts* and *Gcn5* concurrently increased the severity of heart phenotype in fruit flies, but the heterologous rescue succeeded only for *hts.* Nevertheless, when this partial success is corroborated with cardiac phenotypes reported for the equivalent *KAT2B^F307S^* and *Gcn5^F304S^* alleles and the RNAi results, the perspectives are encouraging. It is reasonable to conclude that various interactions between *ADD3* and *KAT2B* variants in patients can be mirrored by interplays of *hts* and *Gcn5* alleles in *D. melanogaster*, helping experts to develop novel drugs able to restore the normal cardiac phenotype.

Intriguingly, a closer inquiry revealed that *Gcn5^WT^* rescues lethality and the morphology of wings, legs and eyes of hemizygous *Gcn5^null^* flies, but, similar to *Gcn5^F304S^* transgenic individuals, these organisms have a smaller diastolic diameter as compared to control flies. A possible explanation for this phenotype is that *Gcn5^WT^* transgene is not in its natural genomic environment where its regulators reside. Nevertheless, when compared to the *Gcn5^WT^* rescued flies, the *Gcn5^F304S^* transgenic ones exhibit supplemental cardiac impairments as reduced contractility and a more irregular heartbeat. This case is a very interesting one, as it shows that subtle phenotypes may still be present even when a complete intraspecific rescue is reported. It seems that sporadic complete phenotype rescue results may remain partial to some degree, as subtle mutant phenotypes may be difficult to notice unless specifically searched for, as exemplified in the study of Gonçalves et al. [160].

Fundamental questions emerge when considering that *KAT2A^WT^* and *KAT2B^WT^* counterintuitively fail to rescue hemizygous *Gcn5^null^* flies, pointing to a functional divergence. Why are *KAT2B^F307S^* and *Gcn5^F304S^* equivalent alleles associated with similar cardiac phenotypes in humans and flies, suggesting an inter-specific functional conservation? Should one always expect that a structural gene orthology is concluded by heterologous rescue experiments? Why does functional complementation of hemizygous *Gcn5^null^* flies with human WT alleles fail? Considering that most heterologous rescue experiments reported for *D. melanogaster* were performed using the GAL4-UAS system, it is helpful to consider the recent work of Casas-Tintó et al. [162]. Due to carefully designed experiments, the authors conclude that the expression of enhancer-Gal4 constructs may be transiently ectopic and influenced by the genomic insertion site. Added to the fact that not a complete human gene sequence but a human cDNA, without regulatory sequences, is usually cloned in a UAS vector, we presume that the unstable activation history of some enhancer-Gal4 constructs may interfere with the heterologous rescue results. 

### 3.3. Other Cardiac Disorders

Other cardiac disorders have been modelled in *D. melanogaster*, such as channelopathies [163,164,165,166,167] and different syndromic [168,169,170] or nonsyndromic cardiomyopathies [171,172,173,174,175,176,177]. To our knowledge, currently, none of these diseases benefit from effective human allele-based functional complementation studies, although some groups successfully tackled the heterologous rescuing of fly cardiac phenotype, the alleles of choice being of animal origin, mainly from mice [173,178,179]. For example, Gao’s group reversed the effect of loss of fly *γ-sarcoglycan* (*Scgδ*) by using a murine counterpart *gamma-sarcoglycan* (*Sgcg*) [179]. An engineered form of the *Sgcg* (termed Mini-Gamma) has been introduced into flies, and it efficiently rescued the cardiac phenotype of an amorphic allele of *Scgδ*. Mini-Gamma was generated by removing a portion of extracellular domain of *Sgcg* that contained a large frameshift deletion, which led to a premature stop codon. Exon skipping corrected the reading frame, expression of Mini-Gamma in the heart tube being sufficient to restore cardiac function to wild-type magnitudes.

A gene involved in cardiac dysfunction independently from canonical Wnt signaling is *pygopus* (*pygo*), which maintains normal heart physiology in aging *D. melanogaster* [180] and is involved in the differentiation of intra-cardiac valves [181] of the fruit flies. Knockdown *pygo* mutant allele underpins cardiac arrhythmias and decreased contractility with systolic dysfunction in fruit flies [182]. The cardiac impairments determined by knockdown *pygo* allele in *D. melanogaster* resemble increased incidence of atrial fibrillation in senior humans. Although *Pygo1* and *Pygo2* are not essential for heart function and development in mouse, they may be involved in preventing senescence phenotypes specific for aging hearts in mammals [180].

An experiment of interest would be the functional rescue of the cardiac phenotype of *pygo* knockdown fruit flies with the transgenic *Pygo1^WT^* allele, a plausible scenario, as the lethality of *pygo^130^*-null embryos was rescued by both *PYGO1^WT^* and *hPYGO2^WT^* human alleles [182]. Again, the experimental paradigm is that if heterologous rescue of a specific severe phenotype such as lethality is possible, then rescuing subtle mutant phenotypes determined by the same orthologs is reasonably plausible. 

The use of high-throughput sequencing techniques and wide-ranging cardiac gene panels dramatically increased the detection of variants of uncertain significance (VUS) [183,184,185,186], whose definite classification requires additional studies including functional ones. The previously presented data, as well as data from Table 2, demonstrate the structural and functional homologies between fruit fly and human cardiac genes and advocate the use of *D. melanogaster* system as a prime candidate to study and validate genetic variants associated with cardiac disorders.

## 4. Cancer

Cancer is a multifactorial and multistep disease characterized by uncontrolled proliferation of tumor cells that escape the control of physiological growth sentinels, apoptosis defects and metabolic alterations. These signaling pathways are conserved in *D. melanogaster*, making the fruit fly an appropriate model organism to study cancer biology [192,193,194,195]. Important processes such as genomic instability, strategies to evade apoptosis, telomerase reactivation, tumor-promoting inflammation and evasion from the immune system, angiogenesis, anaerobic glycolysis, competitiveness of cancer stem cells, importance of tumor microenvironment, invasiveness and metastasis, cancer cachexia, drug screening and resistance have been extensively studied and modeled in *D. melanogaster* [196]. Several examples are provided in Table 3 and the following subchapters.

Epithelial cancer is the most studied type of cancer on the *D. melanogaster* model, as revealed by the high number of citations recorded in FlyBase. This is at least partially explained by the fact that the fruit fly larval imaginal discs, which are morphologically and biochemically comparable to mammalian epithelia, could be used to model different processes involved in the epithelial cancer onset and progression. The imaginal wing and eye discs have been successfully used to study tumor growth and invasion, investigate the function of cancer genes, analyze oncogenic cooperation and perform chemical screenings [205,206,207,208,209].

### 4.1. Validating Orthologs of Human Tumor Suppressors Using the Drosophila melanogaster Model 

In *D. melanogaster*, three complexes are involved in regulating cell growth and differentiation: Crumbs/Stardust/PATJ/Bazooka, Par6/aPKC (atypical protein kinase-C) and Scrib/Dlg/Lgl (Scribble/Discs large/Lethal giant larvae) complexes [210]. The *lgl* was the first neoplastic tumor suppressor gene discovered in *Drosophila*, whose loss leads to an abnormal development (disruption of cell polarity and tissue architecture, uncontrolled proliferation and tumor growth) of the imaginal structures and the larval brain. The apical–basal polarity loss in epithelial cells often occurs in human epithelial cancer, facilitating invasion and metastasis, and therefore a more aggressive profile of the malignancy [211]. Following their transplantation into wild-type recipients, the *lgl* mutant imaginal tumorous tissues could migrate and metastasize in other regions of the fruit fly body, killing the host, thus resembling the human secondary cancers [212]. Other features shared with human metastasis are represented by the upregulation of type IV collagenase and NDP kinase in *lgl-*induced tumors [213,214]. Mammalian homologues of the *lgl* gene (*HUGL-1/*Llgl1 and *HUGL-2*) are highly conserved in humans, highlighting their role in cell growth and initiation of neoplastic lesion. From the two human homologues of the *lgl* gene, the *HUGL-1* LOF has been reported in different types of human cancer (e.g., breast, melanomas, prostate, ovarian and lung cancers) and *HUGL-1* rescued all the defects of the fly *lgl* mutant. For the rescue experiments, the null allele *lethal(2)gl4* has been used. The flies homozygous for the mutant allele are headless pharate, with the eye imaginal disc structure completely lost in the third instar larval stage [215]. The insertion of the *HUGL-1* cDNA in the homozygous mutants led to a partial development of rudimental eyes and larval structures comparable to wild type. The ubiquitous expression of *HUGL-1* in *lgl*-null fruit flies assured the recovery of viable phenotypes (viable adults or completely developed pharate). Despite being completely sterile, they did not develop neoplasia during their lifespan and showed normal imaginal structures, compared to that of the wild-type adults. These results demonstrate that *HUGL-1* can act as a tumor suppressor in *D. melanogaster* and thus represents the functional homologue of *lgl* [197].

### 4.2. Elucidating the Role of Tumor Microenvironment and Host-Neoplastic Cells Competition in Gut Adenoma Development

It is largely accepted that the tumor microenvironment plays an important role in the tumor’s progression, exhibiting either pro-growth or inhibitory effect on the proliferation and invasiveness of neoplastic cells [216]. Cancer cells use cell competition as a form of interaction within the tumor microenvironment [217,218]. Cell competition was first described as a quality control mechanism in *Drosophila* defined as the ability of wild-type cells to kill the mutant cells harboring reduced fitness and growth potential [219]. However, normal cells could also be killed by tumor mutant cells called supercompetitor cells, leading to the development of hyperplasia and adenomas in the adult *Drosophila* midgut [220]. Studies in the *Drosophila* model have demonstrated that cooperation between the tumor suppressor *adenomatous polyposis coli* (*APC*) gene and *wingless* (*Wnt*) is involved in cell competition. Two APC proteins, APC1 and APC2, with different domains and tissue distribution are shared by mammals and *Drosophila*. Fly APC1 and APC2 functions are partially redundant in regulating *Wnt* signaling and cytoskeletal reorganization [221].

As also reported for humans, the *APC* inactivation in *Drosophila* leads to very high levels of *Wnt* target gene expression in different tissues, including the intestine. Akin to the mammalian intestine, *Drosophila* adult midgut epithelium cells have a high turnover rate maintained by the intestinal stem cells (ISCs). Therefore, they have been used as a model to elucidate the role of different signaling pathways in adenoma formation and the role of different mutations in tumor development [222,223,224,225,226]. Loss of function of *APC* leads to abnormal proliferation of ISCs, followed by the loss of gut epithelial cell polarity, hyperplasia and epithelial overgrowth [227,228]. On the other side, the *Wnt* genes are expressed at very high levels in colorectal tumors harboring mutations in *APC*. This proves that upregulation of *Wnt* expression in human ISCs is associated with adenoma development [229]. Importantly, loss of *APC1* leads to the activation of Wnt signaling in retinal photoreceptors, inducing their ectopic apoptosis. Thus, the *APC1* mutant eye phenotype could be used to investigate the roles of *Wnt* signaling pathways in health and disease [230,231]. Using this *Drosophila* model, it has been shown that the *Wnt* pathway genes’ expression in *Drosophila* is regulated by transcription cofactors such as *earthbound1* (*ebd1*) and *erect wing* (*ewg*) [200,232]. From these, *ebd1*, which harbors Centromere Protein B (CENPB) DNA binding domains, is essential for the *Wnt*-dependent control of ISC proliferation. Using the *APC1* mutant eye phenotype, it has been demonstrated that *ebd1* heterozygotes induce a partial suppression of *APC1* mutant apoptosis, the rescue being nearly complete in *ebd1* homozygotes. The human homologue of *ebd1* (*JRK/JH8*) is overexpressed in several carcinomas including colon, breast and ovarian serous cystadenocarcinoma, and has been proven to be associated with elevated expression levels of *Wnt* target genes in human colorectal tumors. *ebd1* and its human ortholog share structural and functional similarity and interact directly with Arm/β-catenin associated in a ternary complex with T cell factor (TCF) [233]. The human Jerky has rescued the flight muscle defects in *ebd1* as well as in *ebd1* and *ebd2* double mutants proving its functional equivalence to Ebd1 and Ebd2 [200].

### 4.3. Demonstrating the Species-Dependent Pathways of Notch Hyperactivation

Coiled-coil and C2 domain-containing protein (CC2D) 1A and 1B are members of the Lgd protein family, conserved among metazoans, with many partially redundant functions such as centrosomal cleavage, molecular signaling, innate immunity response (by modulating TLR3, TLR4 and RLR pathways) and synapse maturation [234,235]. The *D. melanogaster* ortholog *Lgd*, a tumor suppressor gene, has been shown to be involved in Notch signaling. Notch is a conserved developmental signaling pathway involved in essential cellular processes such as differentiation, pattern formation, cell-cycle progression, morphogenesis, migration, apoptosis, T cells activation etc., which is dysregulated in many cancer types [236]. The Notch signaling occurs upon the direct contact between a signal-receiving and a signal-sending cell, mediated by the binding of Delta/Serrate/LAG-2 (DSL) ligand to the trans-membrane Notch receptor. Upon binding, the extracellular domain cleavage allows the release of active receptor Notch intracellular domain (NICD), which accumulates in the nucleus and regulates downstream genes in concert with other proteins [237,238]. Loss of function of *lgd* in fruit flies led to constitutive ligand-independent activation of Notch in epithelial cells and to tissue hyperplasia [239,240,241,242,243,244,245]. However, this effect was not confirmed in mouse epithelial gut, suggesting that *lgd* genes are not involved in the Notch pathway hyperactivation in mammals. In humans, NICD nuclear accumulation was associated with increased tumor cell growth and cell survival and treatment failure in different types of malignancies such as breast, lung and pancreatic cancer [246,247,248,249]. The functional homology between human *CC2D1A* and *CC2D1B* and the fly *lgd* was demonstrated in rescue experiments. Among the two orthologs, one copy of *CC2D1B* was sufficient to completely rescue the mutant [201]. Moreover, the expression of *CC2D1A* and *CC2D1B* is under the control of the endogenous promoter of *lgd* [244]. 

## 5. Infectious Diseases

The *D. melanogaster* model provides a valuable tool for studying the molecular mechanisms of different infectious diseases, the processes involved in anti-infectious immunity and for pharmacological screenings, as thoroughly detailed in the recent review of Harnish et al. [246]. This is at least partially because many infectious agents often modulate highly conserved innate immunity pathways such as the Nuclear Factor kappa B (NF-κB) and c-Jun N-terminal Kinase (JNK) signaling, phagocytosis and apoptosis, which are also present in the fruit fly. We will present below some examples illustrating that *D. melanogaster* represents an appropriate experimental model to recapitulate Koch–Evans postulates [247] regarding the reproduction of human phenotypes for some infectious diseases.

### 5.1. Molecular Mechanisms of Neuropathological Effects Caused by Zika Virus NS4A Protein 

Zika virus is an emerging mosquito-borne flavivirus closely related to Dengue and West Nile viruses [248]. Zika infection is associated with severe neurological symptoms and sequelae, such as Guillain–Barre syndrome and congenital microcephaly [249,250]. These neurological complications are explained by the interaction of the nonstructural 4A (NS4A) protein of Zika virus with ankyrin repeat and LEM domain containing 2 (ANKLE2), encoded by a gene associated with autosomal recessive microcephaly in humans [116,251]. Indeed, the *ANKLE2* heterozygous mutations in humans are associated with infants’ severe microcephaly and later cognitive, neurological, intellectual and developmental deficits [252]. Interestingly, *Ankle2* is involved in brain development in flies, inhibiting the neuroblast division in the third instar larval brain; the hypomorphic mutants (*Ankle2A)* have been proved to be pupal lethal and exhibited small brain volume phenotype [116,253,254].

The *D. melanogaster* model was used to demonstrate the physical interaction between Zika *NS4A* and human *ANKLE2* and its pathological consequences. The ectopic expression of Zika *NS4A* in the developing third instar larva brains of *Drosophila* provoked a reduction in brain lobe volume, induced apoptosis and reduced neuroblast proliferation. This mutant phenotype was rescued by wild-type *ANKLE2* but not by a microcephaly-associated *ANKLE2* variant (*ANKLE2*^Q782X^) [255]. The expression of *NS4A* in fruit flies heterozygous for a hypomorphic allele of *Ankle2* caused a more significant microcephaly in comparison to the condition induced in wild-type fruit flies [255]. Moreover, *Ankle2* regulates the function of genes that control cell polarity during asymmetric division of neuroblasts, including *lethal (2) giant larvae* (*l(2)gl*), *atypical protein kinase C* (*aPKC*), *bazooka* (also known as *par-3*) and *par-6* and *VRK1*, for which human orthologs have been described. These genes are involved, both in flies and humans, in neural stem cell self-renewal and production of neurons, while being also related to developmental brain disorders [256]. From these, the human *VRK1* pathogenic alleles are associated with motor and sensory axonal neuropathy and microcephaly [257]. Mutations in the fly homolog of *VRK1*, *ballchen* (*ball*), induced the loss of neuroblasts in third star *Drosophila* larval brain. It has been shown that NS4A, having the same location as Ankle2 in the endoplasmic reticulum and nuclear envelope, could interact with ball (*VRK1*) to regulate brain size in flies. The *NS4A* expression mimicked the influence of the Ankle2-Ball (VRK1) pathway on the aPKC and l(2)gl proteins, which are critical for brain development. The microcephaly induced by *NS4A* expression has been rescued by removing a single copy of *ball* or *l(2)gl*, demonstrating that *NS4A* hijacks the Ankle2-ball (VRK1) pathway, affecting neuroblast division and brain development, leading to microcephaly [258]. 

### 5.2. Elucidating the Molecular Players in the Cytotoxicity of Cholera Toxin

Cholera is an acute diarrheal infection caused by ingestion of food or water contaminated with the spiraled bacterium *Vibrio cholerae*, causing 1.3–4.0 million cases and 21–143 thousand deaths each year [259]. The most important virulence factor of *V. cholerae* is cholera toxin, a typical AB toxin with ADP-ribosylating action. Cholera toxin stimulates cAMP production in the gut epithelial cells, generating the hypersecretion of water and electrolytes responsible of the specific clinical symptoms such as aqueous diarrhea and rapid, severe dehydration [260]. 

*Drosophila* model was used to investigate the molecular mechanisms of cholera toxin active (A) subunit. For this purpose, the gene for cholera toxin A subunit (CtxA) was expressed in the developing fly wing, causing a CtxA-dependent weight-loss phenotype. The mutant phenotype was fully rescued upon co-expression of an active form of Notch or wild-type Rab11, the most well-represented members of the Ras superfamily GTPases, involved in intracellular vesicle trafficking and signaling [261], and was significantly worsened when a dominant-negative form of Rab11 or Gαs was co-expressed. The *CtxA* expressed in the midgut epithelial cells affected the intestinal epithelial permeability, as revealed by the occurrence of gradual wasting and the smurfing phenotype after flies feeding with food dyed with FD&C blue dye#1 [262]. These phenotypes have been also rescued by co-expression of Rab11, suggesting that cholera toxin A subunit inhibits Rab11-mediated vesicle trafficking. 

### 5.3. Molecular Mechanisms of Apoptosis Induced by Helicobacter pylori Cytotoxin-Associated Gene A 

*Helicobacter pylori* infection affects about 50% of the human population and around 7% will develop gastroduodenal disease [263]. *H. pylori* is associated with various gastric pathologies, ranging from peptic ulcer to gastric adenocarcinoma and lymphoma [264]. The symptomatic infections are produced by strains harboring the *cytotoxin-associated gene A* (*cagA*), one of the main virulence factors [265]. Once delivered in the host cells, CagA is activated through phosphorylation by Src-family kinases and binds to SHP-2, a protein phosphatase encoded by the *PTPN11* gene in humans. SHP-2 further activates signaling pathways downstream of receptor tyrosine kinases (RTKs), a class of receptors with a pivotal role in cancer invasion and metastasis [266,267]. By interfering with epithelial cell adhesion, polarity, migration and differentiation, CagA triggers malignant transformation of gastric epithelial cells, its oncogenic potential being confirmed in transgenic animals [268,269].

The ectopic expression of CagA in the epithelial cells of the *D. melanogaster* developing wing has been shown to induce a dose-dependent apoptotic effect, leading to significantly lower size wings. This phenotype was similar to that produced by the localized activation of the JNK pathway within the wing cells. The cagA-induced apoptosis was suppressed by co-overexpressing a dominant-negative form of *Basket* (*Bsk*), a *Jun amino-terminal kinase (JNK) homolog*, and enhanced by co-overexpressing of wild-type *Bsk* [270], suggesting that CagA is an important mediator of the activation of JNK signaling pathway during *H. pylori* infection [271,272].

Since JNK signaling is dependent on the *Ras* oncogene, it has been further investigated whether CagA can genetically interact with the constitutively active oncogenic variant of *Ras* called p.G12V or *Ras^V12^* [272,273]. If the expression of *Ras^V12^* alone in the fly eye has been shown to induce the formation of non-invasive tumors, its co-expression with *CagA* causes invasive tumors. In addition, *CagA* has been shown to interact with *dlg1* and *l(2)gl*, which are considered neoplastic tumor suppressor genes [274]. 

### 5.4. Discovering Novel Candidates for Assessing Genetic Susceptibility to Different Infections

The *malvolio* (*mvl*) gene of *D. melanogaster* encodes a protein sharing a high homolog to natural resistance-associated human macrophage proteins (Nramps), which are integrated in the phagolysosomal membrane and function as cation transporters [275,276,277]. In *D. melanogaster*, *mvl* is expressed in macrophages and in differentiated neurons. The loss-of-function mutations lead to taste behavior defects caused by a reduction in the sensitivity of the gustatory circuits to stimuli. The ubiquitous expression of human *Nramp-1* protein in mutant fruit flies can fully rescue the taste defect. Moreover, the taste behavioral defects can be suppressed when the fruit flies are grown on media supplemented with Fe^2+^ or Mn^2+^ for a minimum of 2 h before testing, sustaining the role of mvl in the transport of bivalent cations [278].

The polymorphisms of the *Nramp-1* gene have been linked to susceptibility to tuberculosis and leprosy in human populations; therefore, the fact that human *Nramp-1* can fully complement the defect in *mvl* makes *D. melanogaster* an attractive *in vivo* model system for *Nramp-1* functions in different human infections [279,280]. 

### 5.5. Demonstrating the Functional Homology of Human Vasodilator-Stimulated Phosphoprotein (VASP) and Drosophila enabled

*Drosophila enabled* (*ena*), a dominant genetic suppressor of mutations in the *Abelson* (*Abl*) tyrosine kinase, is a member of Ena/human vasodilator-stimulated phosphoprotein (VASP) protein family, which is associated with actin filaments and focal adhesions. Ena is a specific substrate for Abl and also interacts with the SH3 domain of Abl [281]. Among its physiological roles, VASP interacts with *Listeria monocytogenes* Act A protein, which is required for the internalization of this facultative intracellular pathogen, mediating the reorganization of actin filaments. The Ena/VASP domain 1 (EVH1) and EVH2 share 58% and 31% homology between *Drosophila* ena and human VASP, respectively. Moreover, EVH1 is similar to the WP1 domain found in Wiskott–Aldrich syndrome protein, which is associated with cytoskeletal defects in T cells and platelets [282].

Using *Drosophila* model, it has been demonstrated that VASP rescues the lethal phenotype associated with *ena* LOF. The lethal *ena* mutant alleles which affected EVH1 and EVH2 and their capability to bind the focal adhesion protein zyxin and the Abelson kinase were characterized by cytoskeletal defects. A comparison between VASP and three *ena* transgenic lines, tested for their ability to rescue the *ena* null lethal mutants, showed that VASP partially rescued *ena* mutant lethality, 25–85% survival rate and normal development four weeks after eclosion as compared to 79–100% in case of *ena* transgene. Thus, VASP represents a functional substitute of *ena*, the two proteins also sharing the same subcellular distribution and the same expression pattern in mammalian cells. They are detected at the level of actin filaments and focal adhesion contact where they bind to zyxin and the SH3 domain of Abl. Finally, lethal *Ena* mutations have been identified in the most conserved domains of the Ena/VASP family. Further research of this protein family might provide new insights into the regulation of cytoskeleton changes during physiological and infectious processes [283]. The interaction of *Listeria monocytogenes* ActA with VASP may explain the activation of actin reorganization and bacterial cell internalization.

## 6. Discussion

We emphasize that an a priori validation of the functional orthology of an hGOI/dGOI pair by heterologous rescue should be the experimental paradigm for *in vivo* modeling of an HGD. This preliminary approach, even when partially successful, validates the evolutionary conservation of the matching genes and qualifies the respective HGD as an appropriate candidate to be modeled on the *D. melanogaster* platform [13].

Various experimental assays pointing to functional orthology sometimes offer rather indirect data. Although conceptually overlapped, modeling of a human genetic disease by expressing human variants and equivalent fruit fly alleles in *D. melanogaster* does not equate with heterologous rescue experiments. Sometimes, modelling data are not even supported by functional complementation data, as in the case of the *KAT2B/Gcn5* gene pair, described above [160].

We consider that whenever a mutant phenotype of *D. melanogaster* is presumably determined by a new mutant allele relevant for modeling an hGD, an intra-specific rescue with the wild-type copy of dGOI is recommended to confirm this link. There is always a potential risk that the mutant phenotype might have been induced by an unknown mutation, most probably residing on the same chromosome. In such case, the respective mutant strain is not appropriate for heterologous rescue experiments.

A positive heterologous rescue reveals that a basic interactome required for normal functions of the orthologous proteins was conserved between the two species. This aspect favors modeling of the disorder mainly when a chemical rescue is to be further attempted. Targeted mutagenesis of an appropriate dGOI often results in impaired fruit flies mimicking mutant phenotypes specific for the respective hGD. These mutants offer an excellent perspective for understanding the genetic mechanisms fueling the disorder since they are ideal to be subjected to chemical rescue assays, namely testing of candidate drugs on them. Nevertheless, care should be taken when the chemical rescue is efficient in mutant fruit flies impaired by disease-associated fly or human alleles without a preliminary successful heterologous rescue, since conclusions may be wrong. The candidate chemical may not target the mutant protein encoded by dGOI^LOF^ but rather a direct or a close interactor of it. Therefore, it was the adjustment of the stereo-chemical interaction with the defective protein that led to heterologous rescue instead of a repairing process. If the respective interactor is different or absent in mammals, the positive effects of the putative drug may not be reproducible in human patients.

Both a partial rescue and the rescue failure may be explained by either a less effective or an impaired activity of the associated human protein of interest (hPOI) in the context of fruit fly proteome [13]. When heterologous rescue fails, there are a few alternative explanations, and they should be considered before concluding that the two genes are indeed not functional orthologs. Most probably, the mammalian proximal interactome has changed during evolution, therefore that hGOI is *per se* unable to fit into a fruit fly genetic pathway. Therefore, the hGOI may be involved in similar biological processes in mammals but via different molecular avenues. This situation is expected to hinder the extrapolation of some promising chemical rescue results from fruit fly to a mammalian organism. Alternatively, hidden mutations in the genetic background of the fruit fly strain subjected to heterologous rescue may impede the functional complementation results. In this case, it is worthy to outcross the LOF allele in a new, isogenized background and repeat the rescue phenotype experiment. Finally, either the molecular construct used for embryo injection or the GAL4 driver may have inhibitory behaviors, hence alternative approaches may need to be tested [162].

However, there is also a reverse of the medal, namely when one is performing a promising heterologous rescue experiment and does not obtain the functional complementation of the mutant fruit fly. We further discuss various hypothetical scenarios for successful and failed heterologous experiments, just to emphasize the complexity of data interpretation when hGDs are modeled on *D. melanogaster*.

### 6.1. Issues When Modeling hGDs in D. melanogaster Regardless of Positive Heterologous Rescue Results

**Figure 2:**
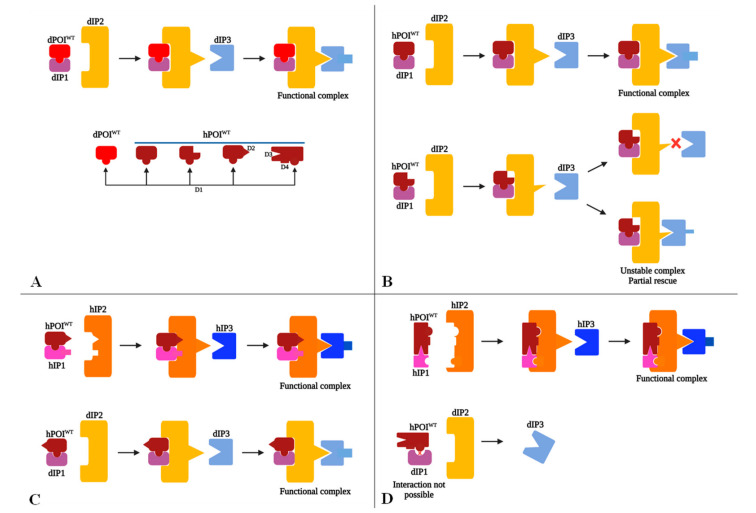
Scenarios for issues of heterologous rescue results. (**A**) *D. melanogaster* wild-type protein dPOI^WT^ encoded by a dGOI has a local interactome, represented by the interacting proteins dIP1, dIP2 and dIP3. When these four proteins interact correctly, the resulting functional complex supports a normal phenotype (**top**). The functional domain of dPOI^WT^ is D1, which is also present in different hPOI^WT^ equivalents (**bottom**); some of these equivalents have additional functional domains D2 and D3. (**B**) hPOI^WT^ allows heterologous rescue (**top**) or has a steric conformation leading to a loose interaction with dIP2. This condition either prevents formation of the functional complex (no heterologous rescue) or leads to an unstable complex, which determines a partial heterologous rescue result. (**C**) The normal pathway in humans involves supplemental interactions among hPOI^WT^, hIP1 and hIP2 (**top**); D1 of hPOI^WT^ may rescue the phenotype (**bottom**), but if D2 is defect in patients, chemical repair of D1 in fruit flies drives to a positive heterologous result which cannot be extrapolated to patients. (**D**) A distinct way to ensemble the functional complex in humans, when the interactome was not evolutionary conserved (**upper**). hPOI^WT^ has supplemental domains D3 and D4; in this case, D4 domain sterically prevents the proper interaction of D1 with dIP1, therefore heterologous rescue intrinsically fails (**bottom**). Created with BioRender.com (accessed on 23 February 2022).

In order to consider a mammalian genetic disorder to be modeled in *D. melanogaster*, a preliminary bioinformatics analysis of both nucleotide and amino acid sequences should be performed. Some scenarios concerning stereo-chemical interactions among an hPOI^WT^ and its proximal interacting proteins which are prone to affect the results of heterologous rescue experiments are summarized in Figure 2.

Let us presume that a structurally orthologous dGOI is identified in the *D. melanogaster* genome and the human and fruit fly proteins share a common functional domain D1 (Figure 2A). Particularly, there may be also present an unknown functional domain D2 in the human protein (Figure 2A), which is not yet characterized and therefore is not described in the specific databases. In addition, let us assume that research papers refer to D1 as being affected in many of the reported patients, therefore, a research project is launched to model the respective disease in *D. melanogaster*. Nevertheless, in some patients, both D1 and D2 may be distorted, but we expect that there is no medical focus on D2 yet.

To construct the model strains, the fruit flies are subjected to targeted mutagenesis of dGOI. Further genetic and molecular analysis inquiry may reveal that the mutant phenotype of adult fruit flies, which mirror medical conditions, are caused by a distorted D1, and the LOF flies are rescued by hGOI^WT^. Candidate chemicals are then tested on the impaired flies and one potential drug compensates the steric distortion of D1, resulting in a partial or complete phenotype rescue of the fruit flies. However, unexpectedly, when this putative drug is tested in mutant mammals, there is no phenotype rescue, because hPOI^WT^ interacts with the equivalent human interacting protein 1 (hIP1) and hIP2 in a different manner in mammals, involving also distinct domains of D2 of hPOI^WT^ and hIP1 (Figure 2C). The drug tested on *D. melanogaster* was able to chemically rescue only the functional domain D1, which is homologous with D1 of hPOI^WT^ but not the conformation of D2. Therefore, a new project is required to understand functions of D2 and then to identify a different drug capable to rescue its spatial conformation.

A special situation is encountered when IP1, IP2 and IP3 are present in both flies and humans, but the hPOI^WT^/hIP1/hIP2 complex is organized by different interactions. The heterologous rescue should not work since, in humans, D1 of hPOI^WT^ is required along with D3 and D4 domains to interact with hIP2 in order to ignite coupling of hIP3 (Figure 2D). Steric constraints caused by D4 prevent formation of hybrid hPOI^WT^/dIP1 and functional complementation fails, although the targeted D1 is conserved between *H. sapiens* and *D. melanogaster* (Figure 2D).

In this case, a preliminary, negative heterologous rescue test suggests that modeling of the respective genetic disorder is more appropriately performed in a mammalian experimental model such as mouse.

### 6.2. Possible Scenarios Accounting for Heterologous Rescue Failure or Partial Rescue in D. melanogaster

Let us consider that a protein complex dPOI^WT^ (*Drosophila* POI^WT^)/dIP1 (*Drosophila* IP1) interacts with dIP2. The resulting complex dPOI^WT^/dIP1/dIP2 activates dIP3, which is a key prerequisite for the normal functioning of a biological process (Figure 2B). The orthologous gene employed for heterologous rescue of specific LOF fruit flies encodes for an hPOI^WT^, for which bioinformatics reveals the presence of the required domain that allows a proper interaction with dIP1. The particular stereochemistry of the hybrid hPOI^WT^/dIP1 complex, however, may result in a loose interconnection with dIP2 (Figure 2B). As a consequence, the hPOI^WT^/dIP1/dIP2 complex is unstable and even unable to activate dIP3. Therefore, the impaired biological process of LOF fruit flies is either partially rescued or not rescued at all by the human orthologous gene (Figure 2B).

The partial rescue results are still an incentive for modeling hGDs [13], but care should also be taken to the message send by an incomplete rescue result. Namely, does a partial rescue result resemble the case depicted in Figure 2B (bottom) or was the complete rescue phenotype impaired by genetic modifiers already present in the genetic background of a particular fruit fly strain? The roles of genetic modifiers as enhancers and suppressors in either precipitation or postponing the onset of symptoms should always be considered, as they represent the core of personalized medical genetics.

Complete heterologous rescue experiments are more encouraging when starting to model an hGD on *D. melanogaster*, but what about the ideal scenario when the orthologous gene of fruit fly rescues the mammalian mutant phenotypes? The orthologous gene pair *atonal* (*ato*, from fruit flies) and *Math1* (from mouse) are both involved in the development of the nervous system of each species, and null alleles were reported for both genes. In an impressive experiment, transgenic *Math1^WT^* allele saved fruit flies with *ato* null phenotypes. Nonetheless, a transgenic *ato^WT^* allele copy also rescued lethality of *Math1* null mice, otherwise unable to initiate breathing movement after birth; the heterologous rescued mice survived to adulthood [104].

Such examples of functional complementation, when mammalian or even other vertebrate orthologs save the fruit fly phenotypes, deserve a strong consideration if the respective gene is associated with an hGD.

## 7. Conclusions

Interdisciplinary research teaming up experts in genetics, bioinformatics, genomics and other medical domains strongly relies on *D. melanogaster* to model both mechanisms and treatment attempts of several impacting hGDs, such as neurological and cardiac disorders, cancers and infectious diseases.

Data emerging from functional complementation assays are very valuable for understanding the contribution of mutations associated with a genetic disorder in the context of an individual genetic background. In this context, carefully designed heterologous rescue experiments are a powerful tool to tackle defiant medical conditions *via* genetic avenues kept open by the *D. melanogaster* experimental model. The functional complementation assay is of prime relevance not only for medical use but also for fundamental research. What are these experiments telling us by revealing that many mammalian genes still function in the molecular context of the ancient *D. melanogaster* genome? Perhaps, this spectacular journey back in time evidences that numerous gene functions are conserved under an inherent selective pressure force: fundamental biological processes are articulated by using the same old molecular tools.

Obviously, some other medical challenges, such as aging issues, are prone to be modeled in *D. melanogaster*, but heterologous rescue assays’ data are still expected for specific orthologs genes. Although diseases such as Hutchinson–Gilford progeria syndrome are figured in fruit fly [284,285], we found no functional complementation experiments reported so far, neither in FlyBase nor in a recent review [286] for genes involved in aging pathology.

Hence, we conclude that preliminary heterologous rescue assays should serve as the standard for genetic analysis of hGDs on the *D. melanogaster* model.

## Figures and Tables

**Table 1:** Successful examples of heterologous rescue experiments related to neurodegeneration. Within the vertebrate gene column, (h) indicates a human gene while (m) stands for a mouse gene. Unless otherwise indicated, WT alleles are implicitly considered. HR is the acronym for heterologous rescue and indicates that the references designate HR studies.

Clinical Impact	Vertebrate Gene	Fly Gene	Mutant Phenotype (Fly)	Heterologous Rescue	HR References
motor neuron diseases	(h)*VAPB*	*Vap33*	loss of Vapp33 determines larval lethality, with few adult escapers	expression of (h)*VAPB* alleviates the lethal phenotype determined by loss of *Vap33*	[68]
Huntington’s disease	(h)*UCP2*	*UCP5*	expression of mutant Huntingtin protein in glia determines altered locomotor performances and uncommon vulnerability to mechanical stress	co-expression of (h)*UCP2*	[69]
	(h)*HTT*	*htt*	*htt*-null flies have severe thorax muscle loss and accelerated deterioration in mobility and lifespan	(h)*HTT* rescues the *htt* loss associated phenotypes	[70]
PD	(h)*MIC60*	*MIC60*	*MIC60^mut^*-null allele determines pupal lethality in homozygous individuals; *MIC60^mut^*/+ flies are normal	expression of (h)*MIC60* in *MIC60^mut^*/+ flies provides a normal phenotype, while expression of mutant (h)*MIC60^A4V, T11A or C17F^* leads to severe adult lethality and reduced larval crawling	[71]
	(h)*PRKN*	*park*	*park-*null flies exhibit reduced lifespan, locomotor and fly defects, infertility, lower cell size and number, progressive degeneration of certain DA neurons	co-expression of (h)*PRKN* rescues the neurotoxicity; muscle-specific expression of (h)*PRKN* rescues the flight ability	[72,73]
	(h)*LRRK2*	*Lrrk*	*Lrrk*-null mutants elicit autophagy defects and DA degeneration	overexpression of (h)*LRRK2* rescues the mutant phenotype	[74,75]
	(h)*VPS35*	*Vps35*	downregulation of *Vps35* in brain determines supernumerous neuroblast phenotype	expression of (h)*VPS35* fully rescues the brain tumor phenotype exhibited by *Vps35* mutants	[76]
PD; frontotemporal dementia	(h)*MAPT*	*tau*	loss of *tau* determines lethality; deletion of *tau* in neurons determines neurodegeneration	expression of (h)*MAPT* partially rescues the neurodegenerative phenotype	[77]
ALS	(h)*PFN1*	*chic*	RNAi-mediated downregulation of *chic* in motor neurons determines pupal lethality	the *chic* mutant phenotype is rescued by expressing (h)*PFN1* in motor neurons	[78]
ALS and other neurodegenerative diseases	(h)*VCP*	*TER94*	*TER94* mutations determine tubular lysosome dysfunction	expression of (h)*VCP* rescues the phenotype determined by mutant *TER94*	[79]
late-onset AD	(h)*TM2D3*	*amx*	strong neurogenic phenotype when *amx* is maternally mutated	(h)*TM2D3* is able to partially rescue the neurogenic phenotype and embryonal lethality	[80]
Parkinsonism with spasticity, X-linked; intellectual developmental disorder, X-linked, syndromic, Hedera type	(h)*ATP6AP2*	*ATP6AP2*	*ATP6AP2* depletion is lethal; RNAi knockdown of *ATP6AP2* in wing pouch leads to abnormal wing development and growth defects	expression of (h)*ATP6AP2* in *ATP6AP2* RNA1 background rescues the specific mutant phenotype	[81]
pediatric-onset neurodegenerative disorder	(h)*ADPRHL2*	*Parg*	*Parg* LOF determines decreased survival in response to oxidative challenge	lethality is rescued by expressing (h)*ADPRHL2*	[82]
neurodegeneration	(h)*TARDBP*	*TBPH*	*TBPH*-null mutants experience loss of the ventral nerve cord neurons (bursicon neurons)	expression of (h)*TARDBP* rescues the bursicon neurons	[83]
neurodegeneration; cancer; metabolic disorder	(h)*TOMM70*	*Tom70*	*Tom70*-null mutation conducted to pupal lethality	the lethality is rescued by the expression of (h)*TOMM70*	[84]
neurodegeneration; Boucher–Neuhäuser, Gordon Holmes, Laurence–Moon and Oliver McFarlane syndromes	(h)*PNPLA6*	*sws*	the *sws^1^*-null mutation causes locomotion deficits and neurodegeneration	(h)*PNPLA6* rescues the mutant *sws* phenotype	[85]
			the *sws^1^* mutants showed characteristic vacuoles in central brain and optic lobes	(h)*PNPLA6* partially rescues the vacuolization of mutant *sws*	[86]
pantothenate kinase-associated neurodegeneration	(h)*PanK2*	*fbl*	a hypomorphic mutation in *fumble* results in flies that have brain lesions, defective neurological functions and severe motor impairment	the paralysis and impaired climbing activity are rescued by expressing (h)*PanK2*	[87]
in mice, neonatal lethality, slow progressive neurodegeneration, enhanced limb-clasping reflexes, impaired motor activity, cognitive deficits and hypomyelination [88]	(h)*NRD1*	*Nrd1*	LOF allele causes neurodegeneration	expression of (h)*NRD1* rescues the pupal lethality and electroretinogram defects	[89]
chorea-acanthocytosis, neurodegeneration, progressive loss of cognitive and locomotor functions	(h)*VPS13A*	*Vps13*	mutant flies have age-linked neurodegeneration and reduced lifespan	overexpression of (h)*VPS13A* in mutant flies rescues the characteristic phenotype	[90,91]
Alkuraya-Kucinskas and Oliver Mcfarlane syndromes	(h)*DENND4A*	*Crab*	flies lacking *Crab* activity experience age-dependent decline in photoreceptor function and structural integrity	expression of (h)*DENND4A* rescues the eye defects exhibited by the mutant flies	[92]
neurodegenerative encephalopathy	(h)*TBCD*	*TBCD*	projection neurons expressing *TBCD^1^* mutant allele have affected axonal branches	overexpression of (h)*TBCD* extensively suppresses the axonal mutant phenotype	[93]
neuronal K^+^–Cl^−^ cotransporter; epilepsy	(h)*SLC12A5*	*kcc*	*kcc^DH1^* hypomorphic allele acts as a seizure-enhancer mutation and exacerbates the bang-sensitive paralytic behavior	(h)*KCC2* rescues the mutant phenotype induced by *kcc^DH1^*	[94]
progressive myoclonus epilepsy	(h)*GOSR2*	*membrin*	homozygosity for *membrin*-null allele causes larval lethality	expressing (h)*GOSR2* fully rescues the larval lethality, but the adults, although normal looking, display severe motor impairments	[95]
early infantile epileptic encephalopathy (EIEE)	(h)*ACTL6B* (*BAF53B*)	*Bap55*	mutations in *Bap55* affect the synaptic connections in olfactory neurons	(h)*ACTL6B* rescues the mutant phenotype of *Bap55*-null individuals	[96]
photosensitive epilepsy (PSE)	(h)*SGMS1*	*-*	*cpes*-null mutants show compromised ceramide phosphoethanolamine synthase and fail to complete neuronal cell body encapsulation in the neuronal cortex	expression of (h)*SGMS1* rescues the PSE and cortex glial aberrations	[97]
ASD	(h)*TaoK2*	*Tao*	loss of *Tao* determines overgrowth of dendritic branching and behavioral defects	(h)*TaoK2* restores the aberrant dendritic branches to control levels	[98,99]
	(h)*DAT* (*SLC6A3*)	*DAT*	*DAT* KO flies are hyperactive	*DAT* KO flies expressing (h)*DAT* have reduced locomotion	[100,101]
	(h)*SCAMP1,* (h)*SCAMP5*	*Scamp*	*Scamp*-null flies exhibit shortened lifespan, compromised climbing, heat-induced seizures and compromised learning and long-term memory	both (h)*SCAMP1* and (h)*SCAMP5* rescue the climbing mutant phenotype; (h)*SCAMP1* significantly improves the learning index of *Scamp*-null flies	[102]
autism; multiple myeloma	(m)*Nbea*	*rg*	*rg*-null mutants exhibit aberrant associative odor learning, modification of gross brain morphology and of synaptic architecture	the transgene (m)*Nbea* is able to rescue only aversive odor learning and synaptic architecture	[103]
affected development of distinct cell types in the central nervous system and in sensory systems	(m)*Math1*	*ato*	mutant *ato* embryos lack precursor cell selection and chordotonal organ specification	expression of (m)*Math1* under the control of the *ato* embryonic enhancer	[104]
			in mouse, (m)*Math1*-null animals do not succeed to initiate respiration and die soon after birth	replacing (m)*Math1* coding region with *ato* allowed the animals to survive to adulthood	
CMT type 2A, axon degeneration [105]	(h)*MFN1*, (h)*MFN2*	*Marf*	mutant flies have affected mitochondria and, as a consequence, their nerves cannot send out signals to muscles; in addition, Marf is lost in the ring gland affecting the production of a hormone required for larva transition to adult, the mutants dying in their larval stage	expression of both (h)*MFN1* and (h)*MFN2* is necessary for hormone production and the rescue of all phenotypes	[106]
CMT neuropathy	(h)*GDAP1*	*Gdap1*	knockdown mutants experience retina and muscle degeneration	the mutant phenotype is rescued by (h)*GDAP1*	[107]
dominant-intermediate CMT neuropathy	(h)*YARS*	*TyrRS*	RNAi-silenced *TyrRS* determines specific bristle phenotypes	expressing (h)*YARS* rescues the abnormal bristle phenotype	[108]
CMT neuropathy type 2D	(h)*GARS*	*GlyRS*	*GlyRS*-null flies lack dendritic and axonal terminal arborization	(h)*GARS* rescues the arborization defects in *GlyRS*-null flies	[109]
autosomal recessive cerebellar ataxia	(h)*UBA5*	*Uba5*	*Uba5*-null mutants have reduced lifespan and locomotor activity as well as neuromuscular junction (NMJ) defects	(h)*UBA5* expression significantly rescues the NMJ mutant phenotype	[110]
FA	(h)*FXN*	*fh*	*fh* mutants have altered mitochondrial functions and exhibit age-dependent neurodegeneration	expression of (h)*FXN* rescues the neurodegeneration	[111]
ataxia determined by defects of autophagy	(h)*ATG5*	*Atg5*	flies lacking *Atg5* activity are unable to walk and fly properly	(h)*ATG5* restores the mutant flies’ normal movements; (h)*ATG5^E122D^* slightly improves the defective mobility	[112]
X-linked Snyder–Robinson syndrome	(h)*SMS*	*Sms*	*Sms* mutants have critically lowered transcript levels that reduce viability	(h)*SMS* rescues the viability of mutant flies	[113]
Delpire–Mcneill syndrome	(h)*SCL12A2* (*NKCC1*)	*Ncc69*	*Ncc69* mutants reach adulthood but their abdominal nerves are swelled and form bulges	this neuropathy is rescued by (h)*SCL12A2*	[114,115]
microcephaly; Zika virus target	(h)*ANKLE2*	*Ankle2*	mutations in *Ankle2* can lead to loss of peripheral nervous system organs in adults and severely reduced brain size in hemizygous third instar larvae	expression of (h)*ANKLE2* rescues the mutant phenotype	[116,117]
neural network formation; tumor progression	(m)*Bsg*	*Bsg*	mutations in *Bsg* alter the cell architecture and can lead to high embryo or larval lethality	*Bsg* LOF in adults’ eyes determines mislocalization of photoreceptor nuclei, a phenotype rescued by expressing (m)*Bsg*	[118]
global developmental disorders, intellectual disability	(h)*CAPZA2*	*cpa*	*cpa*-null allele determines first instar lethality	(h)*CAPZA2* rescues the lethal phenotype of *cpa-*null individuals	[119]
autosomal recessive, nonsyndromic intellectual disability	(h)*ZC3H14*	*Nab2*	*Nab2*-null flies experience developmental and locomotor defects	(h)*ZC3H14* expressed in neurons rescues the *Nab2*-null phenotype	[120]
Troyer syndrome	(h)*SPG20*	*spartin*	loss of *spartin* is associated with motor dysfunctions and brain neurodegeneration	synaptic overgrowth in *spartin*-null flies is rescued by presynaptic expression of Myc-tagged (h)*ZC3H14*	[121]
intellectual disability	(h)*OPHN1*	*Graf*	loss of Graf affects the mushroom body (MB) development	expression of (h)*OPHN1* significantly ameliorates the MB mutant phenotype	[122]
intellectual disability, X-linked	(h)*CASK*	*CASK*	affected expression of *CASK* negatively impacts middle-term and long-term memory	overexpression of (h)*CASK* in neurons of *CASK* mutants fully rescues the memory	[123]
intellectual disability, X-linked	(h)ACSL4	*Acsl*	*Acsl* mutants exhibit neuromuscular junction overgrowth	expression of (h)ACSL4 rescues the mutant phenotype particular to *Acsl* mutants	[124]
intellectual disability	(h)SMARCA5	*Iswi*	*Iswi* LOF is related to decreased body size and movement in larvae and decreased brain size and locomotor dysfunctions in adults	(h)*SMARCA5* expression rescues the *Iswi* specific mutations	[125]
nervous system developmental defects	(h)*EBF3*	*kn*	homozygous *kn*-null genotype is embryo lethal	(h)*EBF3* rescues the lethality	[126]
autosomal recessive neurologic disorder	(h)*TMTC3*	*Tmtc3*	neuron-specific knockdown of *Tmtc3* rises the incidence of mechanically induced seizures	neuron-specific expression of (h)*TMTC3*	[127]
intellectual developmental disorders	(h)*IQSEC1*	*siz*	loss of *siz* affects the growth cones and causes embryonal lethality	overexpression of (h)*IQSEC1* in WT fly background is toxic; lowered expression of (h)*IQSEC1* in *siz*-null mutants partially rescues the embryonal lethality	[128]
developmental delay, movement disorders and metabolic decompensation	(h)*OGDH*	*Ogdh*	LOF allele is associated with early developmental lethality	the expression of (h)*OGDH* rescues the mutant phenotype	[129]
infantile encephalopathy (lethal)	(h)*DNM1L*	*Drp1*	*Drp1* mutants have altered mitochondrial trafficking and die as larvae	ubiquitous expression of (h)*DNM1L* rescues the lethality	[130]
Schizophrenia	(h)*DTNBP1*	*Dysb*	*Dysb* mutants have compromised memory, elevated climbing activity, abnormal male-male courtship behavior, hypoglutamatergic and hyperdopaminergic activities	pan-neuronal or glial expression of (h)*DTNBP1* rescues various *Dysb* mutant phenotypes	[131]
Pitt–Hopkins syndrome	(h)*TCF4-A*, (h)*TCF4-B*	*da*	*da*-null allele severely impacts the embryonic nervous system development	both (h)*TCF4-A* and (h)*TCF4-B* rescue the mutant embryo phenotype	[132]
neurofibromatosis, type 2	(h)*NF2*	*Mer*	*Mer*-null mutations determine lethality	isoform 1 of (h)*NF2* is able to rescue the lethality of *Mer*-null mutants	[133]

**Table 2:** Successful heterologous rescue experiments related to heart disease. Within the vertebrate gene column, (h) indicates a human gene, while (m) stands for a mouse gene. Unless otherwise indicated, WT alleles are implicitly considered. HR is the acronym for heterologous rescue and indicates that the references designate HR studies.

Clinical Impact	Vertebrate Gene	Fly Gene	Mutant Phenotype (Fly)	Heterologous Rescue	HR References
cardiac dysfunction (postulated), TRiC/CCT complex	(h)*CCT4*	*CCT4*	RNAi-silenced *CCT4* determines pupal lethality and growth defects	overexpression of (h)*CCT4* rescues the mutant phenotype	[187]
lipotoxic cardiomyopathy, ceramide/sphingolipid-related	(h)*DEGS1*	*ifc*	knockout of *ifc* results in larval lethality	(h)*DEGS1* rescues the lethal phenotype of *ifc* null individuals	[188]
congenital heart defect (postulated), *KMT2*-related	(h)*KMT2A* (*MLL*)	*trx*	LOF mutations determines larval to pupal lethality associated with aberrant cuticular patterns	expression of (h)*MLL* partially rescues the cuticular phenotype	[189]
dilated cardiomyopathy 3B	(m)*Dmd*	*Dys*	loss of *Dys* function leads to reduced lifespan, significantly increased heart rate, age-dependent myofibrillar disorganization, cardiac chamber enlargement and impaired systolic function	the mutant phenotype was partially reversed by expression of a truncated (m)*Dmd* which restores the cardiac diameters and function	[173,178]
Noonan syndrome	(h)*PTPN11* (*SHP-2*)	*csw*	*csw* mutations determine zygotic lethality	expression of (h)*SHP-2* rescues the zygotic lethality	[190]
muscle and aortic defects, *ARIH1*-related	(h)*ARIH1*	*ari-1*	*ari-1*-null allele is associated with affected larval muscle, lethality or reduced lifespan in adults	(h)*ARIH1* rescues *ari-1*-related lethality.	[191]

**Table 3:** Positive heterologous rescue experiments related to cancer. Within the vertebrate gene column, (h) indicates a human gene. Unless otherwise indicated, WT alleles are implicitly considered. HR is the acronym for heterologous rescue and indicates that the references designate HR studies.

Clinical Impact	Vertebrate Gene	Fly Gene	Mutant Phenotype (Fly)	Heterologous Rescue	HR References
epithelial cancer	(h)*LLGL1* and (h)*LLGL2*	*l(2)gl*	*l(2)gl*/l(2)gl genotype determines lethality	(h)*LLGL1* partially rescues the homozygous *l(2)gl* lethal phenotype; imaginal tissues do not show any neoplastic features, with *Dlg* and *Scrib* exhibiting the correct localization; animals undergo a complete metamorphosis and hatch as viable adults	[197]
	(h)*HUGL-1*	*lgl*	mutations in *lgl* determine structural defects in larvae	(h)*HUGL-1* expression in the homozygous *lgl* mutants leads to a partial development of rudimental eyes and larval structures comparable to wild type	
	(h)LATS1 and (h)LATS2	*Wts*	developmental defects, lethality in flies	(h)*LATS1* rescues all developmental defects including embryonic lethality in flies	[198]
	(h)*Scrib*	*scrib*	polarity and neoplastic overgrowth defects	(h)*Scrib* rescues the polarity and neoplastic overgrowth defects of *scrib* mutants	[199]
	(h)*JRK/JH8*	*ebd1*	muscle defects in *ebd1 and ebd1/ebd2* double mutants	(h)*JRK/JH8* has rescued the flight muscle defects in *ebd1 as well as in ebd1/ebd2* double mutants	[200]
	(h)*CC2D1A* and (h)*CC2D1B*	*Lgd*	tissue hyperplasia in *Lgd* mutant phenotype	(h)*CC2D1A* and (h)*CC2D1B* rescue the *Lgd* mutant phenotype	[201]
various cancers	(h)*TP53*	*p53*	*p53*-null embryos have high sensitivity to genotoxic stressors such as irradiation	(h)*TP53* partially rescues the embryo liability to irradiation	[202]
acute myeloid leukemia	(h)*MLF1* and (h)*MLF2*	*Mlf*	*Mlf* LOF phenotypes include the decrease in embryonic crystal cell numbers and adult bristle and wing phenotypes	expression of (h)*MLF1* and (h)*MLF2* rescues several *Mlf* LOF phenotypes	[203]
	(h)*CUX1*	*ct*	ct deficient flies exhibit abnormal wing phenotypes	(h)*CUX1* rescues the *ct* mutant phenotypes	[204]
